# Beyond *She* and *He*: A Framework for Studying the Cognitive, Psychological and Social Effects of Gender-Neutral Pronouns

**DOI:** 10.1177/0261927X251346193

**Published:** 2025-06-03

**Authors:** Tiziana Jäggi, Pascal M. Gygax, Sofie Decock, Ute Gabriel, Sarah Van Hoof, Hanne Verhaegen, Chloé Vincent

**Affiliations:** 1 Department of Psychology, 27211 University of Fribourg, Fribourg, Switzerland; 2 Department of Translation, Interpreting and Communication, 26656 Ghent University, Ghent, Belgium; 3 Department of Psychology, Norwegian University of Science and Technology, Trondheim, Norway

**Keywords:** grammatical gender, pronoun, non-binary, mental representations, attitudes, gender-neutral, self-identification, stereotypes

## Abstract

In recent years, gender-neutral pronouns have emerged in different languages. We review current research on their emergence and offer an interdisciplinary framework for studying and understanding gender-neutral pronouns. Our framework is aimed at sparking and guiding interdisciplinary research on gender-neutral pronouns. The framework incorporates (cross-)linguistic and cognitive aspects of the function, use and processing of gender-neutral pronouns in light of the fact that different language systems (i.e., conceptual vs. grammatical gender languages) require different degrees of linguistic creativity in order to expand the word class of pronouns. Furthermore, we link these linguistic and cognitive considerations with research on the social-psychological effects of the use of gender-neutral pronouns, more specifically on how they affect the well-being of nonbinary people with regard to social stigmatization and discrimination (minority perspective) as well as how their attitudes are linked with attitudes on non-binary individuals (majority perspective). Integrating these different aspects, we highlight the current gaps in the literature, make concrete suggestions for future research approaches, and emphasize the importance of interdisciplinary research on this multifaceted topic.

## Introduction

Gender-inclusive language^1^ has been a prominent topic in public debates on social justice and in science since the 1970s, with discussions by theologists on neutral language dating back even earlier (Moulton et al., 1978). These debates have mostly centered on the use of the masculine form as a default value, which has been criticized for reinforcing an androcentric perspective (e.g., Braun et al., 2005; Stout & Dasgupta, 2011). Specifically with regard to pronouns, feminist linguists (Bodine, 1975; Pusch, 1979) have objected against the use of masculine third-person pronouns to persons of any gender, as in example (1), or to a group of mixed gender, as in example (2). They have argued that such forms are not truly generic and produce a male bias, criticism that has been supported by a large body of evidence (e.g., Gastil, 1990; Moulton et al., 1978; Noll et al., 2018; Redl, 2021).

(1) If *a reader* likes this book, *he* may also want to read other books by Ferrante.
(2) *Nathalie_conceptuallyFEM_* et *Simon_conceptuallyMASC_* se promènent au bord du lac, car *ils_MASC_* ont congé auhourd’hui.
  “Nathalie and Simon are walking by the lake, because they have a day off today.”

In the past, guidelines and initiatives to make language more gender-inclusive have mostly been based on binary gender concepts (i.e., woman/man; see Elmiger, 2022a for a collection of guidelines), and have aimed to increase the linguistic visibility of women by specifying feminine forms in texts and thereby reducing gender-based discrimination (e.g., Sczesny et al., 2016). The recent increase in the social visibility of nonbinary people, who identify outside of the gender binary, adds a new layer to the promotion of gender-inclusive language reforms and to the debates surrounding them (e.g., Kotthoff, 2020), in that strategies designed to increase the cognitive availability of women may not work for nonbinary people. In particular, advocates of gender-inclusive language have proposed to introduce gender-neutral pronouns in order to overcome the traditional binary distinctions in third-person pronouns (e.g., Zimman, 2019).

In this paper, we propose an interdisciplinary framework for studying gender-neutral third-person pronouns. In particular, we integrate linguistic and psycholinguistic research with studies in social and differential psychology to approach the cognitive, psychological, and social effects and implications of the use of gender-neutral gender pronouns, especially when referring to, or when used by nonbinary individuals.

Our framework provides a broad structure encompassing cross-linguistic and cognitive phenomena that are linked to the processing of pronouns, emphasizing their importance for gender-inclusive initiatives. We identify factors that influence the emergence and evolution of gender-neutral third-person pronouns in languages that differ in the way gender is expressed. In addition, we examine psychological and social implications of using gender-neutral pronouns for nonbinary people. Finally, we identify gaps in the literature and provide an integrative perspective with concrete options for future research. Our framework emphasizes an interdisciplinary approach to studying and understanding gender-neutral pronouns. It is dynamic, allowing for future research to identify additional factors that may need to be considered. Before we present our integrative framework, we need to define the different types of gender that are relevant to this review, and provide some necessary background on the emergence and characteristics of gender-neutral pronouns.

### Biosocial, Conceptual, and Grammatical Gender

Following Ackerman (2019), we distinguish between biosocial, conceptual, and grammatical gender. *Biosocial gender* refers to a person's gender that is comprised of different gender dimensions such as gender role (i.e., societal gender norms such as communal versus agentic traits which pertain to a person's behavior), gender expression (i.e., how a person presents in terms of clothing, physical appearance, and mannerisms), gender identity (i.e., a person's internal sense regarding their own gender), and biological properties of the body (e.g., chromosomal combination, primary and secondary sexual characteristics, and hormonal status) (Ackerman, 2019). For example, Alex is assertive and takes on leadership roles while also being nurturing and supportive toward friends and family (i.e., gender role). Furthermore, Alex wears mostly androgynous clothing and has short hair (i.e., gender expression). Internally, Alex identifies as woman (i.e., gender identity) and her biological characteristics are considered of the female sex, except for her hormonal status which shows elevated levels of androgens.

*Conceptual gender* refers to the gender that is associated with the meaning of a word and encompasses the closely related concepts *semantic*, *definitional*, or *notional* gender (Ackerman, 2019), which some authors also refer to as *lexical* gender (Gygax et al., 2019). For example, when we use the noun *nun*, we convey the information that the person we are talking about is a woman. Other nouns may be gender-neutral in their meaning, such as the noun *nurse*, but still activate stereotypes (e.g., “nurse” is associated with a woman). As a result, conceptual gender is often conflated with stereotypes and real-life knowledge we have about gender. Compared to biosocial gender, conceptual gender is more categorical and less layered. This means that if someone were to write about Alex from the example above and used the word *woman* to describe her, some information from her biosocial gender would be lost.

*Grammatical gender* refers to a formal linguistic feature that is present in some languages, such as French and German, and absent in others, such as Finnish. When it is present in a language, it has consequences for the grammatical process of agreement. Agreement means that different satellite elements (e.g., article, adjective, pronoun, verb, numeral, or preposition) within a sentence or across a text need to match the grammatical gender category of a noun. Grammatical gender categories can include feminine, masculine, neuter, common, etc. (Hellinger & Bussmann, 2001). For example, French has two grammatical gender categories, feminine and masculine, and articles, adjectives, or verb participles need to match the grammatical gender category of the noun, as can be seen in example (3).

(3) La_articleFEM_ lectrice_nounFEM_ a été captivée_particleFEM_ par le_articleMASC_ nouveau_adjectiveMASC_ livre_nounMASC._
  “The (female) reader was captivated by the new book.”

As illustrated in example (3), grammatical gender is not merely a linguistic (morphosyntactic) category, as it frequently correlates with the conceptual and biosocial gender of the referent (e.g., La_feminine_ lectrice_feminine_, [the (female) reader]) when the referent is animate and especially when it is human (Corbett, 1991). Importantly, languages differ in the extent to which they exhibit grammatical gender. This article will consider these differences focusing on languages that have gender markings on the third-person singular pronoun. Some of these languages require agreement with other grammatical structures in the sentences (e.g., French and German), while others do not (e.g., English) or only in a limited way (e.g., Swedish and Dutch). We refer to the first group of languages as *grammatical gender languages* and the second as *conceptual gender languages* (for a more extensive discussion of grammatical gender dimensions across languages, see Gygax et al., 2019 and the contributions to Hellinger & Bussmann, 2001).

### Nonbinary Gender

In recent years, concepts of gender diversity have increasingly moved away from the hegemonic gender binary (i.e., woman/man), and a new vocabulary has emerged to describe identities beyond the binary (e.g., Richards et al., 2017). An important overarching concept is the term *nonbinary* to refer to a person's gender (e.g., Barker & Iantaffi, 2019; Bornstein, 1994; Richards et al., 2017). In Ackerman (2019)’s framework, nonbinary gender can be used to describe a person's biosocial gender when their gender identity falls outside of the “woman–man” gender binary (e.g., Richards et al., 2017). At the same time, the term allows for many different identities and encompasses people who identify as transgender,^2^ who experience fluidity in their gender identity, or who reject the very notion of gender (e.g., Richards et al., 2017; Zimman, 2019). The same heterogeneity applies to the gender expression of nonbinary people. Despite the diversity within the conceptual definition of nonbinary gender, a common topic that has emerged in research on nonbinary people is their lack of visibility and linguistic representation (e.g., Barbee & Schrock, 2019; Conrod, 2020; Zimman, 2019).

### Gender-Neutral Pronouns

Pronouns are a class of words that replace nouns or noun phrases and refer to people, things, concepts, or places that have been named or are understood in the context. Linguists generally consider pronouns to be closed-class: They are function words, part of a fixed paradigm, used primarily to express grammatical relations, high in frequency, and semantically almost empty (Saxena, 2006). In general, closed-classes are more resistant to change than open-classes, such as nouns and verbs, which more easily admit new members. Nevertheless, closed-classes in general, and pronominal paradigms in particular, may still exhibit changes, often in the long run (for a more extensive discussion in relation to English, see Paterson, 2014).

In this article, we focus on third-person (singular) personal pronouns referring to people, as in the examples (4)–(6).

(4) Anna is currently studying for an exam. She will finish her bachelor's degree in three months.
(5) Andrew is baking a cake. Tomorrow, he will celebrate his daughter's birthday.
(6) This person looks familiar to me. They probably go to the same school as me.

In conceptual gender languages such as English, the common third-person singular pronouns used to refer to people are either female (i.e., *she*) or male (i.e., *he*), reinforcing the gender binary and rendering nonbinary people invisible (Conrod, 2020). In grammatical gender languages, such as French, the gender binary is linguistically reinforced not only by the conceptual meaning of the pronoun but also by the agreeing grammatical structures.

Recently, a series of linguistic innovations have been proposed to remedy grammatical, conceptual, and lexical limitations, depending on how gender is grammatically marked (e.g., Hord, 2016; Zimman, 2019). In many languages, gender-neutral pronouns have been introduced, either in the form of neopronouns or by repurposing existing pronouns (e.g., Hord, 2016). These pronouns can be used in different ways: (1) to either refer generically to people without specifying their gender (i.e., unknown person), (2) to refer specifically to nonbinary people, or (3) to conceal the gender of a specific person (i.e., known person). Examples are summarized in Table 1.

**Table 1.** Different Uses of Gender-Neutral Pronouns in English.

Use	
*Generic use*	
Masculine as generic	If *a reader* is captivated by a book, *he* will want to read other books of that kind. (7)
Inclusive	If *a reader* is captivated by a book, *they* will want to read other books of that kind. (8)
*Specific use (nonbinary)*	
Inclusive	*Sasha* ate at *their* favorite restaurant yesterday. *They* had a great time. (9)
*Specific use (gender-concealing)*	
Inclusive	I had a discussion with *a student* over *their* assignment. I told *them they* needed to respect the deadline. (10)

In grammatical gender languages, such as French, a one-to-one translation of these examples is often not possible because of the grammatical complexities that affect different parts of the sentence, so other linguistic solutions are preferred. For example, in French it is more likely to use the epicene word *une personne_feminine_*[a person] and continue with the pronoun *elle_feminine_*[she] in gender-concealing cases.

In what follows, we present a framework that integrates different perspectives on gender-neutral pronouns. We will focus mainly on gender-neutral pronouns for specific reference to nonbinary people, as their (self-)referential role is specifically interesting in a social psychological context. However, readers should be aware that pronouns are not the only tool needed to render a language more gender-diverse, especially in grammatical gender languages. Expanding on these tools would go beyond the scope of this review.

## An Integrative Framework for Studying Gender-Neutral Pronouns

**Figure 1.**
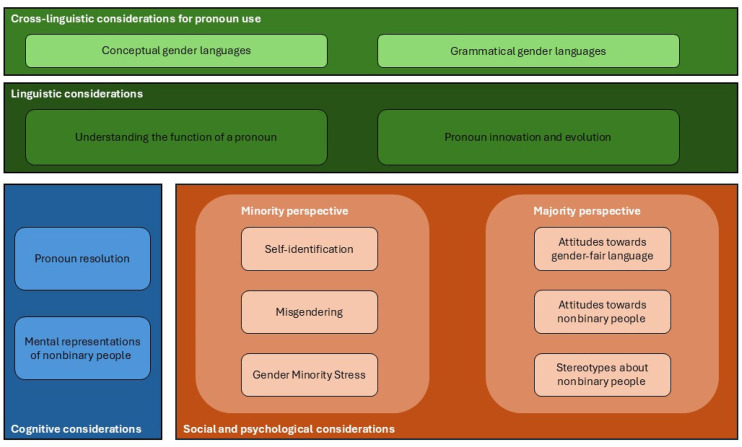
Integrative framework encompassing (cross-)linguistic, cognitive, social, and psychological considerations.

Given the rising cross-disciplinary interest of research on gender-neutral pronouns, our framework (see Figure 1) emphasizes key dimensions that are most crucial in approaching pronoun uses and processing. Specifically, we integrate cognitive, linguistic, social, and psychological dimensions of gender-neutral pronoun understanding and use. While we believe these dimensions can be expanded, we propose that they form the essential foundations for understanding and studying nonbinary pronoun usage.

In the following section, we start with cross-linguistic considerations by describing how different languages differ in the way they grammatically mark gender. We discuss how the linguistic differences pose challenges to the emergence of gender-neutral pronouns. We move to cognitive considerations associated with meaning and gender representations. Finally, we present social and psychological concepts that help understanding and studying gender-neutral pronouns. In particular, we distinguish minority and majority perspectives. To illustrate the key dimensions of our framework, we present not only research that has been carried out but also research that is, to the best of our knowledge, still lacking. Our framework is therefore aimed at sparking and guiding interdisciplinary research on gender-neutral pronoun uses and processing.

### Cross-Linguistic Considerations: Emergence of Gender-Neutral Pronouns in Conceptual and Grammatical Gender Languages

The ease with which certain pronouns emerge to express gender diversity may depend on a language's grammatical, conceptual, and lexical structures. Third-person pronouns are particularly relevant in this respect, as they are marked for gender (masculine or feminine) in many languages. In genderless languages, the notion of gender is not conveyed grammatically, and their third-person pronouns are gender-neutral (Hekanaho, 2021). Nevertheless, even the third-person pronouns in genderless languages can show a male bias rooted in androcentrism, that is the tendency to envision the default human as male (e.g., Bailey et al., 2019; Bailey & LaFrance, 2017; Silveira, 1980). This was demonstrated by Renström et al. (2023), whose participants read a text describing a person in Finnish or Turkish, two genderless languages. Subsequently, the participants had to choose a picture that they believed showed the person they had read about. The participants predominantly selected pictures of men, which indicates that in both languages, the genderless pronouns were male biased. We are not aware of any studies that investigated the cognitive availability of nonbinary people for gender-neutral pronouns in genderless languages.

As explained above, in languages that do show gender-marking on the third-person pronouns, gender-neutral pronouns have been introduced in order to transcend the gender binary. In the following sections, we look in more detail at different gender-neutral pronouns that have been proposed. We will first discuss conceptual gender languages, followed by grammatical gender languages.

#### Conceptual Gender Languages

As pronouns are not accompanied by gender agreement in conceptual gender languages, it is not surprising that gender-neutral pronouns were first introduced in these languages, and that research on these pronouns not only began earlier but is also more extensive (e.g., Hord, 2016). The available studies mostly focus on English and Swedish (for a recent overview, see Renström, 2025), and to a lesser extent on Dutch.

##### The Case of English

In English, the use of so-called singular *they* has long been a subject of debate (Paterson, 2014). The form has for centuries been common for generic reference, gender-concealing reference, and has more recently also been the most commonly proposed form for specific reference to nonbinary individuals.

To explore the specific nonbinary use of singular *they*, Bradley, Salkind et al. (2019) presented participants with descriptions of scholarship applicants using singular *they*. Next, they had to select the person they thought they had read about from a range of photos (representing different gender identities, such as feminine- and masculine-looking persons, but also persons who had a nonprototypical gender expression). The authors found that the responses did not differ significantly from an equal distribution, which shows that singular *they* was mostly interpreted as gender-neutral, that is, also including nonbinary individuals. Further, Bradley, Schmid et al. (2019) found that individual factors, such as personality and attitudes toward gender roles, influenced whether participants considered singular *they* as specifically referring to nonbinary individuals. But not only a person's disposition may influence how singular *they* is interpreted. More recently, Arnold et al. (2021) experimentally investigated whether *they* was interpreted as singular and as specifically referring to a nonbinary individual. They showed that the singular specific nonbinary interpretation was more prevalent in contexts when the protagonist was explicitly introduced as nonbinary (e.g., “Alex uses *they/them* pronouns”). This context dependency of the understanding of singular *they* for nonbinary use was also found in Saguy and Williams (2022)’s qualitative research, which showed that participants could switch from a specific use of singular *they*, as nonbinary, to a generic use of it, depending on the context.

In English, other alternatives, such as neopronouns, have also been coined to refer specifically to nonbinary people. Hord (2016) surveyed the most common gender-neutral pronouns and found singular *they* to be most common, followed by *ze*, *zie* and *xe*. Similarly, Hekanaho (2021) found singular *they* to be most common in their sample, with other neopronouns emerging such as *ze*, *zhe*, *ey*, *e*, or *ae*. This variation in gender-neutral pronouns for nonbinary reference may reflect the need to individualize gender expression and emphasize self-identification through linguistic tools (e.g., Zimman, 2019). Initiatives to prescribe one particular pronoun to refer to nonbinary individuals—as proposed in the *Journal of Transgenderism* (Moser & Devereux, 2019) have met with resistance. Some have argued that as self-identification is an important aspect for nonbinary individuals (Barrett, 2019; Green, 2019), a diversity of alternatives is preferable. Others have also argued, however, that this diversity in the pronouns that are used may prevent them from getting widely accepted by language users (Jones & Mullany, 2019).

##### The Case of Swedish

Swedish provides an example of the successful adoption and wide acceptance of a gender-neutral pronoun. The neopronoun *hen* was already discussed by Swedish linguists in the 1960s (Gustafsson Sendén et al., 2015), was adopted by the LGBTIQ+^3^ community in the 2010s and caught the public eye when it was used in a children’s book in 2012 (Lundqvist & Johansson, 2013).

*Hen* was added to the official guide to the Swedish language, the Swedish Academy Glossary, in 2015. As such, Swedish was one of the first languages to introduce a gender-neutral neopronoun for the third-person singular, which can be used for generic, gender-concealing, and specific nonbinary reference (Gustafsson Sendén et al., 2015). Although *hen* was initially quite controversial among the public, it gradually gained public acceptance in the years following its introduction (Gustafsson Sendén et al., 2015), and its use has now increased dramatically, especially among the younger population (Gustafsson Sendén et al., 2021). Recent studies have shown that *hen* is easy to process, with only a small processing cost visible in the pronoun spill-over regions (i.e., slower reading of the words following the pronoun), which does not seem to impair comprehension (e.g., Vergoossen, Pärnamets et al., 2020).

In another study on the understanding of the pronoun *hen*, Lindqvist et al. (2019) conducted an experiment in which participants read a text about a job applicant who was referred to with different referential strategies: paired forms (*he/she*), the neutral noun phrase *the applicant*, or the gender-neutral pronoun *hen*. After reading the text, the participants had to select a matching photo of the applicant (either women or men). Lindqvist et al. (2019) found that both paired forms and the pronoun *hen* eliminated the male bias that *the applicant* did elicit. They conducted the same study in English and found a male bias for singular *they*, but not for the neopronoun *ze*. The author concluded that newly created pronouns are the best strategy to both avoid the male bias and linguistically include nonbinary people.

To the best of our knowledge, only a few studies (e.g., Renström et al., 2022; Wojahn, 2013) have examined the specific nonbinary uses of Swedish *hen* and its impact on the cognitive availability of nonbinary people. Wojahn (2013), for example, presented participants a text about mobile phone use, which contained either gendered pronouns (*hon* [she]/*han* [he]) or *hen*. After reading the text, participants had to verbally describe the mobile phone user (“I think the mobile phone user is…”). The results showed that when the pronoun *hen* was used, almost one-third of the participants (32%) described a mixed yet binary conceptualization (i.e., women and men). About 28% of participants described a mixed conceptualization that included nonbinary gender (i.e., woman, man, and nonbinary individuals). Interestingly, *hen* was the only pronoun that also generated a specific transgender conceptualization, although only in 4% of participants. Importantly, Wojahn's (2013) findings are based on data collected before *hen* was officially accepted in Swedish. Renström et al. (2022) also found that the generic use of *hen* was preferred to the specific nonbinary one, which they suggested was most likely due to negative attitudes toward nonbinary gender.

##### The Case of Dutch

Dutch is an example of a conceptual gender language that has only recently introduced gender-neutral pronouns. In 2022, the Nederlandse Taalunie (Dutch Language Union), the organization responsible for developing and promoting policy on the Dutch language, recognized several gender-neutral pronouns, with *hen* and *die* and their possessive pendants *hun* and *diens* currently being the most commonly used forms (Decock et al., 2024; Vriesendorp, 2024). Both pronouns already existed in Dutch but with other grammatical functions. The most recent research on the uptake of the Dutch gender-neutral pronouns indicates that the majority of the population are by now familiar with them (Verhaegen et al., under review).

In terms of usage, Vriesendorp (2024) showed that Dutch speakers vary their use of pronouns for nonbinary reference, depending on the grammatical function (i.e., subject, object, or possessive pronoun). For example, participants preferred to use *die* as a subject and *hen* as an object when referring to nonbinary people. Decock et al. (2024) experimentally investigated how these pronouns are perceived in texts. Their participants read a newspaper text about a nonbinary person for whom gender-neutral pronouns were used. Two sets of pronouns were tested, used in subject, object, and possessive functions, respectively: *hen-hen-hun* and *die-hen-hun*. Participants’ comprehension and appreciation of the text were measured. Decock et al. (2024) found that the gender-neutral pronominal combinations *hen-hen-hun* and *die-hen-hun* did not impair perceived text comprehension compared to pronoun avoidance and binary pronouns. Overall, the authors concluded that *die* showed more potential for acceptance as a gender-neutral pronoun than *hen*. In an experiment comparing the perception of gender-neutral *die* for specific nonbinary, and for generic reference, Decock et al. (2025) found that *die* for specific reference was less appreciated that for generic reference. These findings mirrored those of Bradley, Schmid et al. (2019) for English singular *they*, and those of Renström et al. (2022) for Swedish *hen*.

#### Grammatical Gender Languages

So far, to the best of our knowledge, no official and formal language authority has proposed a gender-neutral pronoun in grammatical gender languages. In what follows, we highlight the situation for French and German, as they exemplify the complexity of gender-neutral pronoun in grammatical gender languages.

##### The Case of French

In French, the pronoun *iel*, a fusion between *il* [he] and *elle* [she], has become popular enough to be mentioned in the online edition of the French dictionary *Le Robert* (Le Robert, 2023).

*Iel* was coined as a neopronoun for specific nonbinary reference but has also been adopted for generic reference (Le Robert, 2023). In order to simplify the French language as a whole, Elmiger (2022b) suggested moving away from third-person pronouns based on grammatical gender (i.e., the feminine *elle*, the masculine *il*) to a generic third-person pronoun (i.e., *iel*, or another alternative). However, the author noted that such an option (i.e., a fusion between two binary pronouns) may limit self-identification and self-expression for nonbinary individuals (Elmiger, 2022b). Others have argued that *iel* is suitable for both generic and specific nonbinary reference, whereas neopronouns such as *ael*, *al*, or *ol* (as suggested by Alpheratz, 2018) have been found to be interpreted as referring specifically to nonbinary individuals (Rendl, 2023). Knisely (2020) showed that *iel* was the most comprehensible neopronoun for generic and specific nonbinary reference. The author also acknowledged that many other specific nonbinary pronouns (e.g., *ael*, *al*, *ol* or *al*) are used, which they interpret as illustrating the importance of pronoun diversity for self-identification (Knisely, 2020). Note that most research on the use of these pronouns has been based on corpus studies (Rendl, 2023) and questionnaires (Knisely, 2020), To our knowledge, the only study investigating how these pronouns are processed is Vincent et al. (in preparation), who replicated Decock et al.'s (2024) methodology for the French gender-neutral pronouns *iel* and *al*. Their findings show that participants were significantly more familiar with *iel* (71.3%) than *al* (5.8%). Reading a text with *iel* led to reduced text comprehension and appreciation ratings compared to name repetition, and lower appreciation compared to binary pronouns. This effect was explained by the irritation caused by *iel*. Interestingly, while *al* impaired content recall, *iel* did not.

Importantly, and we will come back to this issue, in grammatical gender languages such as French, where linguistic agreement is central, neopronouns must often be accompanied by other neological constructions to accommodate noun, determinant, adjective, and past participle agreement. For the latter, Alpheratz (2018) suggested for French that all past participles ending in *-é*, *-i*, and *-u* (e.g., *allé* [gone], *sorti* [exited], *revenu* [came back]) become invariable as *-ez*, *-iz* and *-uz* (e.g., *allez* [gone], *sortiz* [exited], *revenuz* [came back]). Knisely (2020) found that contracted forms (e.g., *alle·é* [gone], *sorti·e* [exited], *revenu·e* [came back]) are fully comprehensible in combination with gender-neutral pronouns such as *iel*, yet a specific reference to nonbinary people would require an “x”, as in *iel est revenu·e·x*. The complexity of agreement in languages such as French (and other linguistic constraints) has sometimes compelled nonbinary French-English bilinguals to switch to English, a linguistic strategy referred to as *binary-constrained code-switching* (Kaplan, 2022).

##### The Case of German

In German, most initiatives to advance the use of gender-neutral pronouns have been driven by private stakeholders, such as the Verein für Geschlechtsneutrales Deutsch (Association for Gender-neutral German).

A community-based study conducted by this association with 500 participants found that *dey, hen*, *em*, *sier*, and *en* were the most popular neopronouns (Verein für geschlechtsneutrales Deutsch e.V., 2021). Based on these results, the German feminist magazine *Missy Magazine* decided to introduce the pronoun *dey* for the specific reference to nonbinary people (Vogler, 2022).

So far, the studies conducted in German on neopronouns mainly focused on the use of nonbinary pronouns and surveyed nonbinary and transgender communities (Bloch, 2023; Hord, 2016). Hord (2016) found that German-speaking participants mostly used strategies that avoided the use of pronouns (e.g., rephrasing to avoid pronouns or repeating a person's name) and Bloch (2023) found *they* and *hen* to be the most used pronouns, although the sampled population was very small (*N* = 10).

Overall, different languages have shown great variation and innovation in using and understanding pronouns to refer to nonbinary people, which in turn may have prevented characterizing systematic processes in their development. We would therefore like to offer some linguistic insights to facilitate research in this area.

### Linguistic Considerations

#### Innovation and Evolution of Pronouns

The emergence of gender-neutral pronouns that we described in the previous sections can be regarded as linguistic innovations that follow the stages of linguistic standardization as described by Haugen (1966; also see Deumert & Vandenbussche, 2003). The first stage, *selection,* entails choosing a normative form among several linguistic alternatives. In the second stage, *codification,* the normative form is established in grammars, dictionaries, and style guides. The third stage, *implementation,* consists of the gradual diffusion and acceptance of the newly created norm among speakers—which can succeed or fail.

It is useful to consider these stages when comparing languages with emerging gender-neutral pronouns. It can be argued that conceptual gender languages are most advanced in the standardization of their gender-neutral pronouns. In English and Swedish, for example, gender-neutral pronouns have been codified in authoritative dictionaries and style guides and the implementation stage has been fairly successful, in that people use the pronouns in their everyday lives (e.g., Gustafsson Sendén et al., 2021; Keener & Kotvas, 2023). In Dutch, the pronouns *hen* and *die* have been codified by the Dutch Language Union, dictionaries, and newspaper style guides, but their implementation is still ongoing (Decock et al., 2024). The situation in grammatical gender languages is less advanced and more unstable, and therefore more diverse: For example, in German, the selection process is ongoing and has not yet reached codification (Verein für geschlechtsneutrales Deutsch e.V., 2022), while in French the pronoun *iel* has been codified but is still controversial and widely debated in the public sphere (Conruyt, 2021). We argue that considering the structure of a language is important when researching gender-neutral pronouns, as this structure may influence the implementation and acceptance of these pronouns, independently of social and psychological considerations.

Additional factors may influence the disparity in the development of standardization of gender-neutral pronouns, such as the differences in timing, origin, and range of use. The timing of the introduction varies between languages. For example, the generic use of singular *they* has been common for many centuries (Balhorn, 2004) while the specific nonbinary use was introduced more recently (Bjorkman, 2017). In Swedish, the neopronoun *hen* was introduced in 2015, over ten years ago (Gustafsson Sendén et al., 2015). Therefore, for Swedish, there has therefore been more time to discuss, implement, and study the linguistic innovation compared to more recent introductions such as in French and Dutch. Additionally, it matters whether a newly introduced gender-neutral pronoun is a neopronoun or a repurposed one, as both come with their own advantages and disadvantages (Rott, 2023). It also matters whether the new pronoun strongly resembles the already existing binary pronouns or not. For instance, Swedish *hen* is very similar in form and pronunciation to the established binary pronouns *hun* and *han*, which is often less the case for gender-neutral pronouns in other languages. Their range of use may also play a role in the adoption process: When gender-neutral pronouns are used for all possible types of reference (generic, non-binary, and to conceal the gender of a specific person), as in English and Swedish, they will occur more often from a purely quantitative perspective, which can in turn speed up the habituation process. When they are only used for nonbinary reference, however, without being taken up for generic reference, habituation is likely to take longer.

### Cognitive Considerations

We argue that cognitive considerations are crucial to include in a framework for the study of gender-neutral pronouns. This entails focusing on the underlying mechanisms of processing and interpretation of pronouns by language users. In what follows, we highlight two relevant psycholinguistic concepts: pronoun resolution and mental gender representations.

#### Pronoun Resolution

*Pronoun resolution* is the process of interpreting the meaning of a pronoun in a sentence. Psycholinguistic research has mainly focused on the mechanisms involved in personal pronoun resolution, revealing several overarching principles. First, number agreement is important for pronoun resolution for many languages (e.g., Holler & Suckow, 2016). This means that when one uses a singular pronoun, one expects it to refer to a single person or entity. However, there are also examples of how the meaning of a pronoun—in terms of number agreement—can be more flexible and can change over time. For example, in Early Modern English (i.e., from 1500 to 1800), there were two different pronouns to refer to a single person in the second person (i.e., *thee*) and a group of people in the second person (i.e., *you*). In the first half of the 19th century, the use of *thee* began to decline until the pronoun *you* became the norm for both the singular and plural meanings (Seltzer Krauthamer, 2021). This historical shift shows that despite pronouns being a closed-class, it is possible for plural pronouns to be repurposed as singular. A similar change is exemplified by English *they*, which has become the dominant form for third-person singular generic reference, at the expense of generic *he* (Paterson, 2014).

Second, gender agreement is another important mechanism for establishing the meaning of a pronoun (Schmitt et al., 1999). When the grammatical gender does not overlap with the conceptual gender, agreement can be more ambiguous (Audring, 2013). A common example in German is the word *das Mädchen* [the girl], which is grammatically neuter but conceptually female. As a result, there are two correct ways to establish pronoun agreement, as described in example (11).

(11) German:

- *Das_neuter_ Mädchen_neuter_* spielt draussen. *Es_neuter_* geniesst das schöne Wetter.
  “*The girl* is playing outside. *It* is enjoying the good weather.”
- *Das_neuter_ Mädchen_neuter_* spielt draussen. *Sie_feminine_* geniesst das schöne Wetter.
  “*The girl* is playing outside. *She* is enjoying the good weather.”

In some cases, the mechanisms of number and gender agreement are not sufficient to determine the antecedent, for example, when there are two entities that do not differ in number and gender. In these cases, we use *heuristics*, mental shortcuts that simplify decision making, to resolve the pronoun (de la Fuente et al., 2016). One of these heuristics is the *first-mention bias* (also known as the subjecthood effect; Hwang, 2018). This heuristic guides pronoun resolution to favor the person mentioned in the subject position of the sentence (e.g., Arnold, 2015; Burmester et al., 2018). In example (12), we are more likely to interpret the personal pronoun *she* as referring to Anne rather than Sarah, even though both interpretations would be grammatically correct.

(12) Anne had dinner with Sarah. She intently savored the main course.

When faced with such ambiguous situations, speakers tend to use more explicit referring expressions such as the person's name or a definite description, as in example (13).

(13) Anne had dinner with Sarah. Anne intently savored the main course.

This tendency for explicit lexical expressions instead of pronominal use is called the *gender congruency effect* and is more common in languages that do not have gendered pronouns (e.g., Finnish; Fukumura et al., 2013). Interestingly, these more explicit referring expressions are also used for nonbinary reference. In German, for instance, a common strategy to refer to nonbinary people is to repeat their name (also referred to as the *no pronoun* strategy; e.g., Bloch, 2023). This could indicate that when no norms for nonbinary pronouns have been established, like in German, language users may also avoid ambiguity or uncertainty about the meaning of a pronoun by repeating the person's name.

In Dutch, number agreement and first-mention bias complicate the introduction of and choice between the gender-neutral pronouns *hen* and *die*. Originally, *hen* is a plural object pronoun, and *die* is a demonstrative pronoun. Both have been repurposed as gender-neutral pronouns. While *hen* faces resistance among language users because of the plural association, *die* exhibits less the first-mention bias in the case of more than one antecedent compared to personal pronouns (Decock et al., 2025; Van der Molen, 2023; Van Hoof & Decock, forthcoming). The Dutch gender-neutral pronouns *hen* and *die* thus exhibit differences in terms of pronoun resolution and are not functionally equivalent.

#### Mental Gender Representations

As discussed above, in many languages, the use of masculine forms for generic reference, or masculine generics has long been common (Gygax et al., 2019). Many studies have shown, however, that they have a substantial impact on readers’ mental representations of gender: They mainly generate mental representations of men (or boys). For this reason, different alternative referential strategies have been proposed. One possible strategy is gender specification or differentiation, that is using pair form (e.g., *he or she* in English). Another is neutralization, that is using a gender-neutral pronoun. In English, singular *they* has become the most commonly used form for generic reference.

For generic use, it has been shown that both differentiation and neutralization help to reduce the male bias that is caused by the use of masculine forms (e.g., Gabriel et al., 2018). We argue that this applies to the specific use as well, so considering the mental gender representations that gender-neutral pronouns activate is important to evaluate their cognitive (and social) impact. A common paradigm for assessing mental gender representations is the *sentence-continuation task* (e.g., Gygax et al., 2008), based on Tanenhaus and Carlson’s (1990) *makes-sense* evaluation task. When such a task is used to study mental gender representations, participants read a sentence describing a group of people with a plural role noun (e.g., *the musicians*) or a pronoun. This sentence acts as a prime. Participants then evaluate whether a target sentence, word, or picture, symbolizing the gender of one or several persons, is a good match, or a good continuation of the first one (e.g., “some of the women opened their umbrellas because it was raining” or a picture representing men/women). The task can provide valuable insights into whether the pronoun *he* (as a prime for example) is more likely to be associated with mental representations pertaining to a woman or a man (in a sentence, or in a picture). However, when it comes to investigating what content is more easily associated with nonbinary pronouns, the task may be more challenging, as it is not yet clear what a nonbinary mental representation consists of, both linguistically and symbolically. In fact, researchers have often concluded that when no specific facilitation was apparent for a woman or man continuation (either verbally or choosing pictures), it may have been illustrative of the inclusion of nonbinary individuals (see earlier discussion on the Swedish pronoun *hen*).

As a tentative basis for investigating more precise mental gender representations elicited by the use of gender-neutral pronouns, we first present studies on attitudes toward and stereotypes of nonbinary individuals. As most of these studies have focused on transgender or gender nonconforming people (e.g., Anderson, 2022; Gallagher & Bodenhausen, 2021; Reiman et al., 2023), these will serve as the point of departure for our later research suggestions. For example, Gallagher and Bodenhausen (2021) investigated stereotypes of transgender and cisgender women and men that are prevalent in a cisgender population. The study found that participants agreed more on cisgender stereotypes than on transgender stereotypes, indicating that transgender individuals are less likely to be stereotyped within the traditional gender roles. Furthermore, the participants also displayed distinct patterns of stereotyping for trans- and cisgender women and men, without consistent overlap. This research suggests that different types of stereotypical information can be used to test readers’ mental representations derived from gender-neutral pronouns. In other words, stereotypical information can act as prime, or target, in studies examining mental representations linked to the use of different pronouns.

Gallagher and Bodenhausen (2021) also found that participants with higher scores on gender essentialism held more negative stereotypes of transgender groups and were more likely to stereotype them based on their sex assigned at birth. In a similar vein, Anderson (2022) found that cisgender people's attitudes toward transgender people were influenced by their conceptualization of transgender identity. Those who defined transgender identity as *sickness* or *confusion*, were more prejudiced against transgender people, while those who referred to *transgender identity* as an inherent trait held more positive attitudes towards transgender people. Bi- and homosexual participants were more likely to mention gender identity and less likely to describe transgender people as confused, compared to heterosexual participants.

Insights into mental representations are also provided by studies assessing the visual features of gender stereotypes. Fasoli et al. (2023) examined how participants assigned pronouns, professions and personality to three male, female and androgynous faces (i.e., faces sharing both female and male traits). Overall, the male face was seen as masculine and the female face as feminine, while the androgynous face was seen as rather feminine. Cisgender participants displayed more stereotypical beliefs and used fewer gender-neutral pronouns compared to transgender and non-binary participants. However, the use of gender-neutral pronouns depended on their openness to nonbinary gender, so participants who were more open toward nonbinary gender were more likely to use gender-neutral pronouns. Similarly, Weißflog and Grigoryan (2024) examined gender categorization of faces with cisgender participants and found that providing participants with additional information about profession and behavior was crucial for reaching conclusions when the faces were ambiguous. Overall, around 10% of the targets were rated as nonbinary. Atwood and Axt (2021) used faces to measure implicit attitudes towards androgyny, revealing that these attitudes were influenced by explicit attitudes such as general political ideology and support for nonbinary affirming policies: A stronger preference for gender conforming faces was associated with more conservative political ideology and lower support for nonbinary affirming policies. In all, faces may function as an interesting pathway to participants’ mental representations.

In a nutshell, the mental representations elicited by different gender-neutral pronouns used for specific reference may vary depending on how readers conceptualize nonbinary gender and what attitudes they hold towards it. As such, studies on this topic need to pay particular attention to the specifics of the populations being tested. This is crucial, given that most evidence on this topic suggests that people's conceptualizations of transgender identity are far less consensual than those associated with binary gender roles (e.g., cisgender women are expected to be more communal whereas cisgender men are expected to be more agentic). Further, they may be associated with a person's attitudes toward nonbinary gender.

In a recent study, Zacharski and Ferstl (2023) concentrated on prototypical gender expression. Using pictures, they tested whether the German nonbinary gender asterisk used in contracted forms of role nouns (e.g., Lehrer*in [teacher_man/woman/non−binary_]) could elicit mental representations that also include nonbinary individuals. To do so, they presented pictures of prototypical men, women or nonbinary individuals, for whom participants had to decide whether they were a good match to preceding role nouns presented either in the masculine, feminine, or nonbinary contracted form. Results showed a priming effect of the nonbinary contracted form on all three picture categories, which was not the case when role nouns in the masculine form were used (i.e., there was a male picture preference). To the best of our knowledge, this was the first study to show that a nonbinary linguistic construction (the gender asterisk) can generate representations that are in line with nonbinary gender identity, at least with prototypical pictures of nonbinary individuals.

Renström et al. (2023) conducted a similar study in Swedish and English in which they used *normative* and *non-normative* portraits of people (i.e., stereotypical gender expression versus nonstereotypical gender expression) to evaluate whether paired pronouns (*he/she*) and gender-neutral pronouns (*hen*, singular *they*, *ze*) would evoke a normative gender bias (i.e., overrepresentation of binary gender categories). For Swedish, they observed that the paired pronouns (*han*[he]*/hon*[she]) evoked a normative gender bias whereas *hen* did not. Similarly, for English, they found that *he/she* evoked more gender bias compared to singular *they* and *ze*. However, singular *they* and *ze* only showed no normative gender bias when the instruction clearly stated that these pronouns could be used to specifically refer to nonbinary individuals. A recent study by Van Berlekom et al. (2024) found that the use of the Swedish *hen* increased the representation of nonbinary people in a subsequent gender categorization task suggesting some sort of nonbinary gender mental representations. As the discussion above shows, more research is needed to see whether gender-neutral pronouns manage to accomplish the effect of evoking “balanced” mental gender representations that also include nonbinary people. At the very foundation of this is the understanding of how exactly we (i.e., researchers and participants) conceptualize nonbinary (and binary) gender.

### Social and Psychological Considerations

In the following parts, we focus on social and psychological considerations specific to the link between non-binary gender and gender-neutral pronouns. As such, we find it helpful to distinguish between a majority perspective as well a minority perspective towards nonbinary people. It is important to consider aspects characterizing a majority perspective that influence the acceptance of nonbinary people in our societies, such as prejudice and harmful stereotypes against nonbinary people. These aspects have substantial consequences in the lives of nonbinary people. By applying a minority perspective, we can identify common and unique struggles that nonbinary individuals face in terms of gender-neutral pronouns (mostly caused by the majority perspective), but also how the use of these pronouns may expand to other topics imminent for their health and well-being.

#### Majority Perspective

As discussed earlier, it is easier to move toward gender-neutral pronouns in conceptual gender languages than in grammatical gender languages. Nevertheless, gender-neutral pronouns are topical in societal debates across many countries (e.g., Conruyt, 2021; Vogler, 2022). To the best of our knowledge, no study has systematically compared the discursive arguments on this topic across different countries. However, Vergoossen, Renström et al. (2020) did investigate the discursive arguments against the pronoun *hen* in Sweden. They found that some arguments were related to linguistic aspects, such as defending the linguistic status quo (i.e., “language should not change”), or finding *hen* distracting in communication. Some arguments revolved around the issue of cisgenderism, defined as a specific form of sexism that reveals hostility toward nonbinary individuals (Vergoossen, Renström et al., 2020). Others diminished the issue of gender equality and considered it as irrelevant (Vergoossen, Renström et al., 2020).

It is interesting to consider not only what arguments are put forward against gender-neutral pronouns but also what underlying determinants of a person can be observed in people who oppose these pronouns, or other types of gender-inclusive linguistic structures. Interestingly, the study by Bradley (2020) mirrored the findings of Vergoossen, Renström et al. (2020) on the content of the arguments against *hen,* finding that negative attitudes toward singular *they* were more prevalent among people with sexist beliefs and the belief that it is important to conserve the language as it is. Generally, resistance toward gender-inclusive strategies has been linked to right-wing conservatism (e.g., Sauteur et al., 2023) in that the more right-wing a person is, the more negative attitudes they hold toward inclusive language (and the less knowledge they have about it). Molin et al. (2021) have also linked conservatism (i.e., right-wing ideology) to a lack of openness toward the notion of nonbinary gender. Openness toward nonbinary gender may well be an important factor in explaining people's willingness to accept the use of nonbinary pronouns to refer to a nonbinary person (Renström et al., 2022). Similarly, Patev et al. (2019) found that people with negative attitudes toward transgender people have more difficulty to adopt and use gender-inclusive language in general.

Although resistance to nonbinary gender, or to gender-neutral pronouns, can be linked to conservatism, other factors, such as the strength of one's own binary gender identification and *need for closure* (i.e., need to find clear answers and avoid ambiguity), may also explain some of the resistance (Morgenroth et al., 2021). Age may also be associated to attitudes toward both the notion of nonbinary gender as well as gender-neutral pronouns, in that younger age has been shown to be significantly associated with more positive attitudes (Gustafsson Sendén et al., 2015). This association is weakened as a function of general interest in gender issues. And as mentioned above, Gustafsson Sendén et al. (2021) found that since the pronoun *hen* was introduced in Sweden, attitudes over time became more positive towards the use of *hen.* Since younger people are using *hen* in their everyday lives, the authors consider it likely that *hen* will stay in the language.

Overall, research indicates a strong link between negative attitudes toward gender-neutral pronouns and nonbinary gender identities, highlighting the need for strategies to reduce these negative attitudes. As a consequence, these attitudes lead to the stigmatization and discrimination of nonbinary individuals in our societies. So, addressing harmful stereotypes can foster greater acceptance and usage of gender-neutral pronouns. To tackle this issue, it is beneficial to explore literature on reducing prejudice about transgender and gender-nonconforming people. Broockman and Kalla (2016) showed that a single 10-min conversation, where cisgender participants had to actively take the perspective of a transgender person, could significantly reduce prejudice against transgender people for at least three months. More generally, interventions designed to reduce prejudice for nonbinary people may consult literature on intergroup contact (see Hässler et al., 2021 for a review and model). By addressing these underlying biases, we can adopt a social justice perspective in our research on gender-neutral pronouns.

#### Minority Perspective

Third-person pronouns are used to refer to and represent others, but they are also important for self-identification and self-determination, especially for transgender and nonbinary individuals. Language can be an important gender-affirming measure and is often used as part of social transition (Richards et al., 2017). In fact, gender-neutral pronouns can be considered a way of self-identifying and a strategy for *ungendering* a person's social self (Barbee & Schrock, 2019). As such, researchers should always carefully consider the pronouns that individuals commonly use for self-identification. For a minority population that often suffers from stigma and discrimination (Testa et al., 2015), using the pronoun chosen by a nonbinary person can promote feelings of authenticity, pride, liberation, and confidence (e.g., Barbee & Schrock, 2019; Zimman, 2019). However, due to the risk of discrimination, many nonbinary individuals are cautious to disclose their identity only in safe social contexts (Barbee & Schrock, 2019).

The most common form of linguistic discrimination against nonbinary people is *misgendering*, which occurs when referring to someone using a third-person pronoun or form of address that does not match the person's identity (Ansara & Hegarty, 2013). McLemore (2015) found that misgendering has a negative impact, in that it is associated by transgender people with increased hostility, guilt, and anxiety, as well as a sense of less authentic social interactions, and a perception that transgender people are more stigmatized in society. McLemore (2018) related these findings to the *gender minority stress* model (Testa et al., 2015), in which the amount of internal and external stigma and discrimination a transgender person is confronted with in their everyday lives can impact their mental health. Within this model, misgendering can be subsumed under the distal stressor *nonaffirmation of gender identity* (see Testa et al., 2015 for the model overview). Research on gender minority stress has shown that increased discrimination-related stressors coupled with lower resilience can contribute to poorer mental health (e.g., Budge et al., 2013; Hendricks & Testa, 2012), which explains the higher prevalence of affective disorders in transgender and nonbinary populations (e.g., Reisner et al., 2016). For example, in their study, Jäggi et al. (2018) found that nonaffirmation of gender identity was the most important distal stress factor explaining depressive symptomatology in a transgender population. Recent studies showed that misgendering was similarly impacting nonbinary people (e.g., Flynn & Smith, 2021; Jacobsen et al., 2024). As to how frequent nonbinary people are confronted with misgendering, Jacobsen et al. (2024) found that more than half of their participants (59%) reported misgendering to occur daily and 30% reported misgendering to occur weekly or monthly.

To avoid misgendering, gender-neutral pronouns may be particularly useful. Some studies have even shown that one's use of gender-neutral pronouns increases one's actual acceptance of nonbinary people (Tavits & Pérez, 2019). In addition, misgendering can occur when people prioritize attributing someone's gender even if this may not be the person's actual gender identity, and at the same time ignore that they may be attributing an inaccurate gender to someone (Conrod, 2020). Note that on some occasions, speakers may even deliberately misgender, simply to be deliberately rude or hurtful (Conrod, 2020). Because of the performative act of ascribing gender to oneself (e.g., Kukla & Lance, 2023), misgendering diminishes a person's self-respect, and therefore acts as a psychological microaggression (e.g., Kapusta, 2016). Sevilla Requena (2024) studied deliberate forms of misgendering in online discourse (i.e., tweets on X) directed at transgender and gender-nonconforming communities. Based on their corpus of 400 tweets, they showed that intentional misgendering was connected to the use of further discriminatory language that reflect harmful stereotypes targeting transgender and gender-nonconforming individuals.

In recent years, societal awareness about this issue seems to have increased, in that people have started to introduce their pronouns in certain social contexts, for example, when meeting new people. This pronoun signaling, or sharing, is intended to indicate how one wishes or chooses to be referred to (Zimman, 2019). But *identity signaling* is not the only function of pronoun sharing. Kodipady et al. (2022) found that pronoun sharing is also perceived as *reputation signaling* (i.e., attempting to enhance one's own reputation) and as *norm support* (i.e., sincerely endorsing pronoun sharing as a norm). Importantly, participants rated *identity signaling* and *norm support* as higher when the person sharing their pronouns was presented as transgender than when they were presented as cisgender. In an organizational context, the practice of sharing pronouns was also found to be an effective identity-safety cue for transgender and gender non-conforming persons (Johnson et al., 2021).

However, as is often the case when minoritized groups gain visibility by the societal majority, they are met with political resistance (e.g., Rudman et al., 2012). This political resistance is often referred to as *backlash* (e.g., Vandello, 2025)*.* A current review by Renström (2025) describes how this process starts with the politicization of gender and pronouns, which then leads to a polarization in attitudes toward nonbinary pronouns. Sadly, the backlash extends to the very existence of transgender and nonbinary people, which amounts to higher rates of murdered transgender people and anti-transgender legislation (e.g., Brightman et al., 2024). Vandello (2025) found four different strategies that are relevant when researching transgender backlash: *minimization* (e.g., trivializing issues that are important to the group), *denial* (e.g., denying the existence of a marginalized group), *moralization* (e.g., claiming that the equality efforts of the group are immoral) and *exaggeration of threat* (e.g., claiming that the marginalized group poses threat to majority groups). When researching gender-neutral pronouns, it is important to consider these societal implications from a minority perspective. However, if we care to diminish these threats, research on the majority perspective is as important.

## Quo vadis? Future Research Avenues

**Figure 2.**
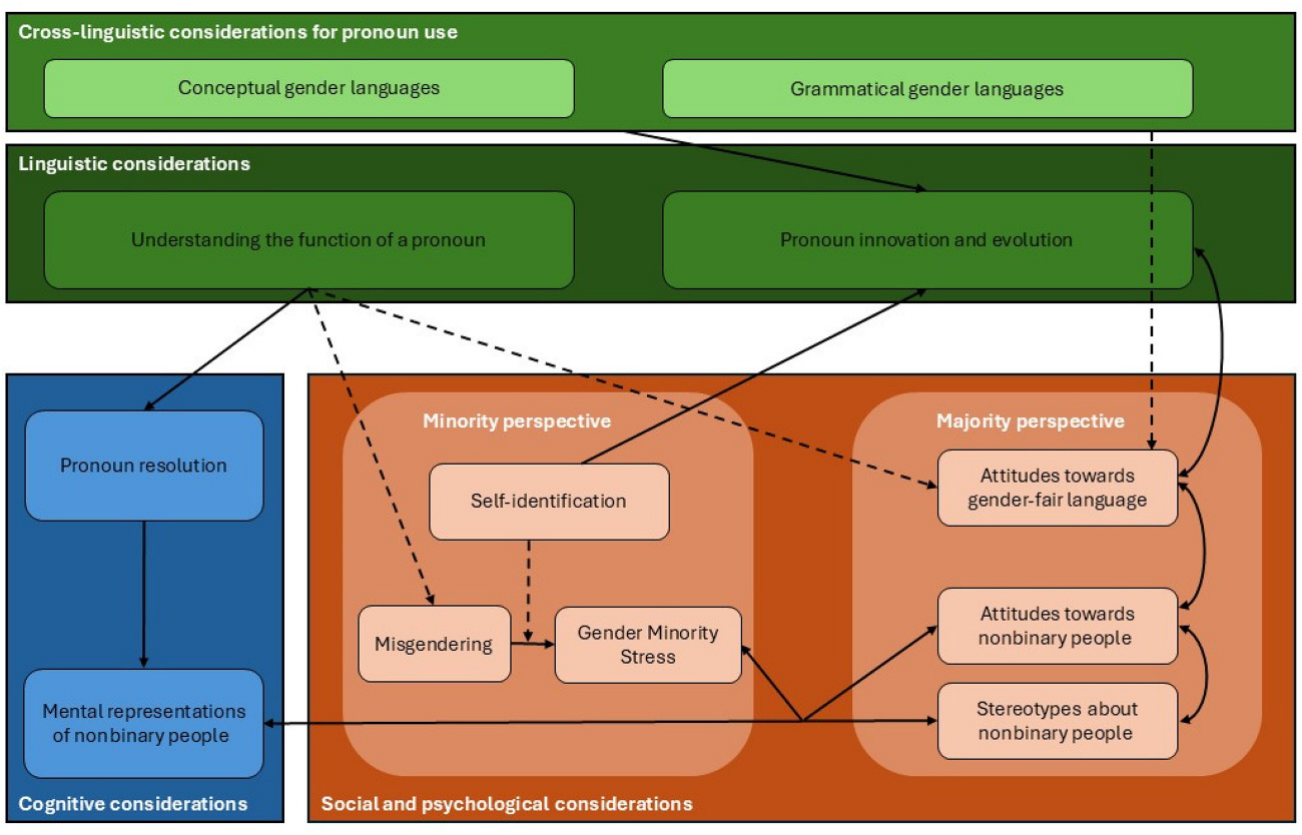
Integrative framework encompassing (cross-)linguistic, cognitive, social, and psychological considerations, as well as relationships between aspects (full arrow) and future research avenues (dotted arrow).

After reviewing the (cross-)linguistic, cognitive, social, and psychological domains for studying gender-neutral pronouns, we extend our framework with arrows symbolizing the connections between these domains. Further, we add dotted arrows to indicate possible research avenues (see Figure 2). In what follows, we demonstrate how the framework can generate significant research questions by presenting five aspects that we consider most important (Figure 2)

First, linguistic considerations may have an important influence on pronominal innovation and evolution. The introduction of gender-neutral pronouns is a recent phenomenon in most languages, occurring almost simultaneously across various countries. Therefore, it would be interesting to study the emergence and implementation, or decline, of these pronouns and, as Renström (2025) has suggested, compare successful and ineffective strategies across languages with different grammatical gender structures. Relevant questions here, among others, pertain to the influence of grammatical gender agreement across different language systems. An associated question is whether agreement influences readers’ and speakers’ mental representations of gender regardless of whether binary or gender-neutral pronouns are used. To the best of our knowledge, agreement in languages such as French has received very little attention in terms of mental gender representations.

Second, the linguistic considerations presented may also be linked to social and psychological considerations. For example, attitudes toward gender-inclusive language may also influence the extent of pronoun innovation and development, which in turn may influence attitudes toward gender-inclusive language (positively or negatively). Some speakers may welcome innovation, have more positive attitudes toward gender-inclusive language, and be more open to the idea of nonbinary gender. Others may perceive linguistic innovations as threatening to their identity and may become increasingly negative towards any form of gender-inclusive language, and in turn, close themselves off to the idea of nonbinary gender. To the best of our knowledge, these relationships have never been studied systematically, nor from a cross-linguistic perspective. The latter might also document the extent to which the complexity of linguistic agreement might play an important role in the general acceptance of gender-neutral pronouns, and thus in attitudes toward gender-neutral language and nonbinary people.

Third, turning to the cognitive considerations, we believe that linking pronoun resolution with mental representations of nonbinary helps us understand how in some instances resolving a pronoun in a text or eliciting a nonbinary mental gender representation may not be successful, either when people have no knowledge about the gender-neutral pronoun used or they do not know the definition of nonbinary gender. Both cognitive considerations are dependent on an overarching understanding of the functions of pronouns, that is whether language users understand why gender-neutral pronouns are necessary for nonbinary individuals. These links may seem trivial but have not yet empirically been investigated. However, the strength of our integrative framework lies in linking cognitive considerations to social and psychological considerations, such as misgendering and stereotyping of nonbinary individuals. Indeed, metalinguistic knowledge about pronoun use may influence the understanding of nonbinary pronouns and may ultimately help to reduce misgendering assuming that some misgendering occurs due to a lack of metalinguistic knowledge about what a pronoun expresses. These questions remain open, of course.

Fourth, in terms of mental representations, and since—as discussed earlier—most gender-neutral pronouns are polysemic in that they have different possible meanings (i.e., generic or specific), future studies need to examine what exactly readers and listeners mentally represent when processing nonbinary pronouns. Previous studies that investigated the impact of other forms of gender-inclusive language (e.g., neutralization and specification strategies in role nouns) on mental gender representations may serve as the basis to build this new research avenue (for a review see Sczesny et al., 2016). These mental representations may be linked to stereotypes about nonbinary people and may be influenced by readers’ and listeners’ attitudes toward nonbinary people. This avenue of research might be especially important to promote nonbinary gender-inclusive language policies, which essentially aim to increase the linguistic visibility of nonbinary people. Some studies have already started to investigate this challenging issue, showing for example that the use of the Swedish *hen* increased representations of nonbinary people in a subsequent gender categorization task (Van Berlekom et al., 2024).

Fifth and finally, future research should include the needs and reflections of nonbinary people. Indeed, most linguistic innovations and their evolution emerge from nonbinary communities, which can guide pronoun preferences in terms of self-identification. Incorporating a minority perspective allows researchers to understand the actual consequences of gender-neutral pronoun and discriminatory form related to pronoun use (e.g., misgender) on nonbinary individuals’ lives. Further, as a vulnerable population in terms of gender minority stress (Jäggi et al., 2018), nonbinary people should be consulted when the goal of a study is to identify language policies to increase their linguistic and societal visibility. As visibility can be a double-edged sword in terms of safety for non-binary people (Barbee & Schrock, 2019; Osborn, 2022), this aspect needs to be considered for different cultural backgrounds, and especially in an increasingly hostile political climate against transgender and nonbinary individuals (Brightman et al., 2024). We would argue that participatory research methods (a mix of qualitative and quantitative methods) may be ideal for this. From a majority perspective, prejudice against nonbinary people should also be reduced to protect nonbinary people from harmful interactions. Future research should identify appropriate methods and interventions, and especially with regard to gender-neutral pronoun, determine which forms of misgendering as well as personal properties in the majority population are malleable to the reduction of harmful stereotypes.

## Conclusion

All in all, we need more research to document the emergence, the use, the interpretations, and the individual and social effects of gender-neutral pronouns. This is important as most languages do not have the linguistic tools to adequately convey the gender diversity encountered in contemporary society, and linguistic innovations are therefore necessary. This needs to be addressed from different angles by engaging in interdisciplinary efforts. Yet, to further develop gender-inclusive language policies and research, researchers should always acknowledge (and communicate with) stakeholders, such as language policy organs, groups of people that are rendered invisible by prevailing language structures, and ordinary language users.

## Notes

1. Gender-inclusive language is sometimes also referred to as *gender-fair language*, or *nonsexist language*, these terms implying to diversifying linguistic expressions to better include gender categories other than the male category. In this article, we use *gender-inclusive language* as an umbrella term encompassing all possible linguistic alternatives to androcentric grammatical or lexical features.

2. Transgender refers to a person whose gender identity does not align with the person’s sex assigned at birth. Its meaning is juxtaposed to cisgender, which means a person’s gender identity aligns with the sex assigned at birth (Drescher et al., 2012).

3. LGBTIQ+ is an abbreviation for lesbian, gay, bisexual, trans, intersex, queer plus more.
